# Machine Learning for the Prediction of Red Blood Cell Transfusion in Patients During or After Liver Transplantation Surgery

**DOI:** 10.3389/fmed.2021.632210

**Published:** 2021-02-22

**Authors:** Le-Ping Liu, Qin-Yu Zhao, Jiang Wu, Yan-Wei Luo, Hang Dong, Zi-Wei Chen, Rong Gui, Yong-Jun Wang

**Affiliations:** ^1^Department of Blood Transfusion, The Third Xiangya Hospital of Central South University, Changsha, China; ^2^College of Engineering and Computer Science, Australian National University, Canberra, ACT, Australia; ^3^Department of Blood Transfusion, Renji Hospital Affiliated to Shanghai Jiao Tong University, Shanghai, China; ^4^Department of Laboratory Medicine, The Third Xiangya Hospital of Central South University, Changsha, China; ^5^Department of Blood Transfusion, The Second Xiangya Hospital of Central South University, Changsha, China

**Keywords:** liver transplantation, machine learning, prediction model, red blood cell transfusion, SHapley Additive exPlanations

## Abstract

**Aim:** This study aimed to use machine learning algorithms to identify critical preoperative variables and predict the red blood cell (RBC) transfusion during or after liver transplantation surgery.

**Study Design and Methods:** A total of 1,193 patients undergoing liver transplantation in three large tertiary hospitals in China were examined. Twenty-four preoperative variables were collected, including essential population characteristics, diagnosis, symptoms, and laboratory parameters. The cohort was randomly split into a train set (70%) and a validation set (30%). The Recursive Feature Elimination and eXtreme Gradient Boosting algorithms (XGBOOST) were used to select variables and build machine learning prediction models, respectively. Besides, seven other machine learning models and logistic regression were developed. The area under the receiver operating characteristic (AUROC) was used to compare the prediction performance of different models. The SHapley Additive exPlanations package was applied to interpret the XGBOOST model. Data from 31 patients at one of the hospitals were prospectively collected for model validation.

**Results:** In this study, 72.1% of patients in the training set and 73.2% in the validation set underwent RBC transfusion during or after the surgery. Nine vital preoperative variables were finally selected, including the presence of portal hypertension, age, hemoglobin, diagnosis, direct bilirubin, activated partial thromboplastin time, globulin, aspartate aminotransferase, and alanine aminotransferase. The XGBOOST model presented significantly better predictive performance (AUROC: 0.813) than other models and also performed well in the prospective dataset (accuracy: 76.9%).

**Discussion:** A model for predicting RBC transfusion during or after liver transplantation was successfully developed using a machine learning algorithm based on nine preoperative variables, which could guide high-risk patients to take appropriate preventive measures.

## Introduction

Liver transplantation is an effective method for treating end-stage liver disease. Prolonged and complicated surgical procedures may cause bleeding during the perioperative period. Most patients require an infusion of concentrated red blood cells (RBCs) during or after the surgery. Although blood transfusion can increase the patient's oxygen supply and improve tissue perfusion, it is also accompanied by many side effects, such as the increased risk of deep vein thrombosis, increased fibrosis, cancer recurrence, and increased mortality, thus adversely affecting the patient's prognosis (1–5). The methods of reducing blood transfusions include the preoperative use of tranexamic acid, intraoperative blood salvage, and intraoperative autotransfusion. However, these approaches cannot be applied to all patients, considering their risks and the costs (6–8).

It is necessary to predict RBC transfusion before the surgery and provide clinicians with practical clinical decision-making guidance. Clinically, physicians make transfusion decisions primarily based on a patient's hemoglobin level and symptoms of anemia. However, other perioperative indicators should not be ignored, for example, essential patient characteristics such as sex, age, and weight; preoperative symptoms such as the presence of portal hypertension, ascites, and hepatic encephalopathy; and preoperative laboratory parameters such as hemoglobin, creatinine, and transaminases. Meanwhile, data on the transfusion of RBCs before surgery and the clinical significance of intraoperative and postoperative risk factors such as operation time, intraoperative blood loss, and postoperative laboratory indicators are limited. Studies have been conducted to predict blood transfusion in joint surgery, craniofacial surgery, and obstetric surgery by developing clinical prediction models combined with patients' preoperative risk factors (9–11).

Machine learning is a field of artificial intelligence that learns from data based on computational modeling. Cutting-edge machine learning models can fit high-order relationships between covariates and outcomes in a vast amount of data. Therefore, they can be applied to complex medical problems and usually perform better than traditional statistical analysis, especially when analyzing big medical data (12). If the RBC transfusion in liver transplant patients can be predicted before surgery, targeted preventive measures are taken for high-risk patients. Unnecessary costs and side effects can be reduced, which is beneficial to the treatment and prognosis of patients. Most studies on predicting RBC transfusion during liver transplantation are based on traditional linear models and logistic regression (LR). However, no studies have been conducted to predict RBC transfusion in patients during or after liver transplantation using a machine learning model (13, 14). Therefore, this study hypothesized that preoperative data from patients could be used to predict RBC transfusion during or after surgery using machine learning.

The purpose of this study was to determine the preoperative risk factors associated with RBC transfusion in patients undergoing liver transplantation and then develop a machine learning model to predict RBC transfusion during and after surgery.

## Materials and Methods

### Study Subjects

The participants were patients aged more than 18 years who underwent liver transplantation, from March 2014 to September 2019, at one of the following three tertiary hospitals: the Second Xiangya Hospital of Central South University, the Third Xiangya Hospital of Central South University, and the Renji Hospital affiliated to Medical College of Shanghai Jiao Tong University. The transplanted livers used in three hospitals were provided free of charge by the Red Cross Society of China. Approval was obtained from the institutional review board for this study (Ref 2019-S007). No written consent was required in view of the purely observational nature of the study. No identifiable data of the donors or live transplant patients were recorded during the whole study.

The commonly used operative methods for liver transplantation currently include the classical liver transplantation (15), piggyback liver transplantation (16), and classical venous bypass liver transplantation (17). Most patients in the three hospitals in our study underwent the piggyback liver transplantation. The major advantage of this method is less intraoperative bleeding (18, 19). Especially for patients with portal hypertension, it can reduce the massive bleeding of posterior peritoneal collateral circulation due to the removal of inferior vena cava (19, 20). Therefore, only patients who underwent the piggyback liver transplantation were included in our study.

Patients who received preoperative blood transfusions and those whose missing rates of data were more than 80% were excluded. Data of patients who underwent liver transplantation from October 2019 to January 2020 were collected prospectively in the Third Xiangya Hospital of Central South University to validate the proposed model further.

### Study Design and Data Collection

A total of 24 preoperative variables were collected within 24 h before the day of surgery. For some variables with multiple measurements, the values closest to the surgery's start time were assessed. The collected preoperative information included patients' demographic characteristics (age and sex), clinical characteristics (weight), diagnosis (cirrhosis, malignant liver tumor, liver failure, alcoholic hepatitis, viral hepatitis, hepatic space-occupying lesions, cholestatic liver disease, or others), preoperative clinical signs (portal hypertension, hepatic encephalopathy, and ascites), and preoperative laboratory indicators (albumin, globulin, and total protein). All variables were obtained from the electronic medical record systems of the three hospitals. Three authors (LL, JW, and YW) had access to the systems and collected the data.

The data collected by different hospitals were converted and unified. For example, 1 mg/dl of creatinine is equal to 88.4 μmol/L. The related variables were combined into one; for example, “hepatocellular carcinoma” and “primary liver cancer” were combined into “malignant liver tumor.” The diagnostic variables were transformed into ordinal variables: 1 = cirrhosis; 2 = liver malignant tumor; 3 = liver failure; 4 = alcoholic hepatitis; 5 = viral hepatitis; 6 = hepatic space-occupying lesions; 7 = cholestatic liver disease; and 8 = others.

### Statistical Analysis

The dataset was randomly split into a training set and a validation set. The data of 835 (70%) patients were used to develop our models, while the data of 358 (30%) patients were used as a validation set.

Continuous variables between transfused and nontransfused groups were compared using either the Student *t*-test or rank-sum test as appropriate. The chi-square test or Fisher's exact test was employed to compare the differences in the categorical variables.

The dataset was imputed using multiple imputation. Then, the recursive feature elimination (RFE) algorithm was used to select key variables and develop a machine learning model named eXtreme Gradient Boosting (XGBOOST) (21–23). In short, RFE is a feature selection method that recursively fits a model based on smaller feature sets until a specified termination criterion is reached. In each loop, features are ranked by their importance in the trained model. By recursively eliminating one feature with the lowest importance, RFE attempts to eliminate dependencies and collinearity that may exist in the model. Features were recursively eliminated until the model's AUROC was <0.80. Then, the last eliminated feature was replaced to make the AUROC more than 0.80. At last, the most important features were screened out, and a XGBOOST model was developed based on the feature set. Other features were not added because they only brought a small increment in AUROC but significantly increased the difficulty of model application.

The proposed prediction model was built in the XGBOOST package in Python language, validation was carried out using the five-fold cross-validation method, and then the AUROC of the training set was calculated. After the model was established, the SHapley Additive exPlanations (SHAP) package in Python was used to explain the model by analyzing two cases. The SHAP package interpreted the output of the machine learning model using a game-theoretic approach (24). For each prediction sample, the model connected optimal credit allocation with local explanations.

Besides, eight other models were developed and compared with the proposed machine learning model, including K-Nearest Neighbors, Naïve Bayes, Support Vector Machine, Multi-Layer Perceptron, Random Forest, AdaBoost and Gradient Boosting Decision Tree, and LR. The validation was also carried out using the five-fold cross-validation, and then the AUROCs were calculated. The sensitivity and specificity were also analyzed.

Finally, the proposed model and the other models were applied to prospective validations. Wrongly predicted samples were analyzed by an experienced clinician and a data scientist.

## Results

**Figure 1:**
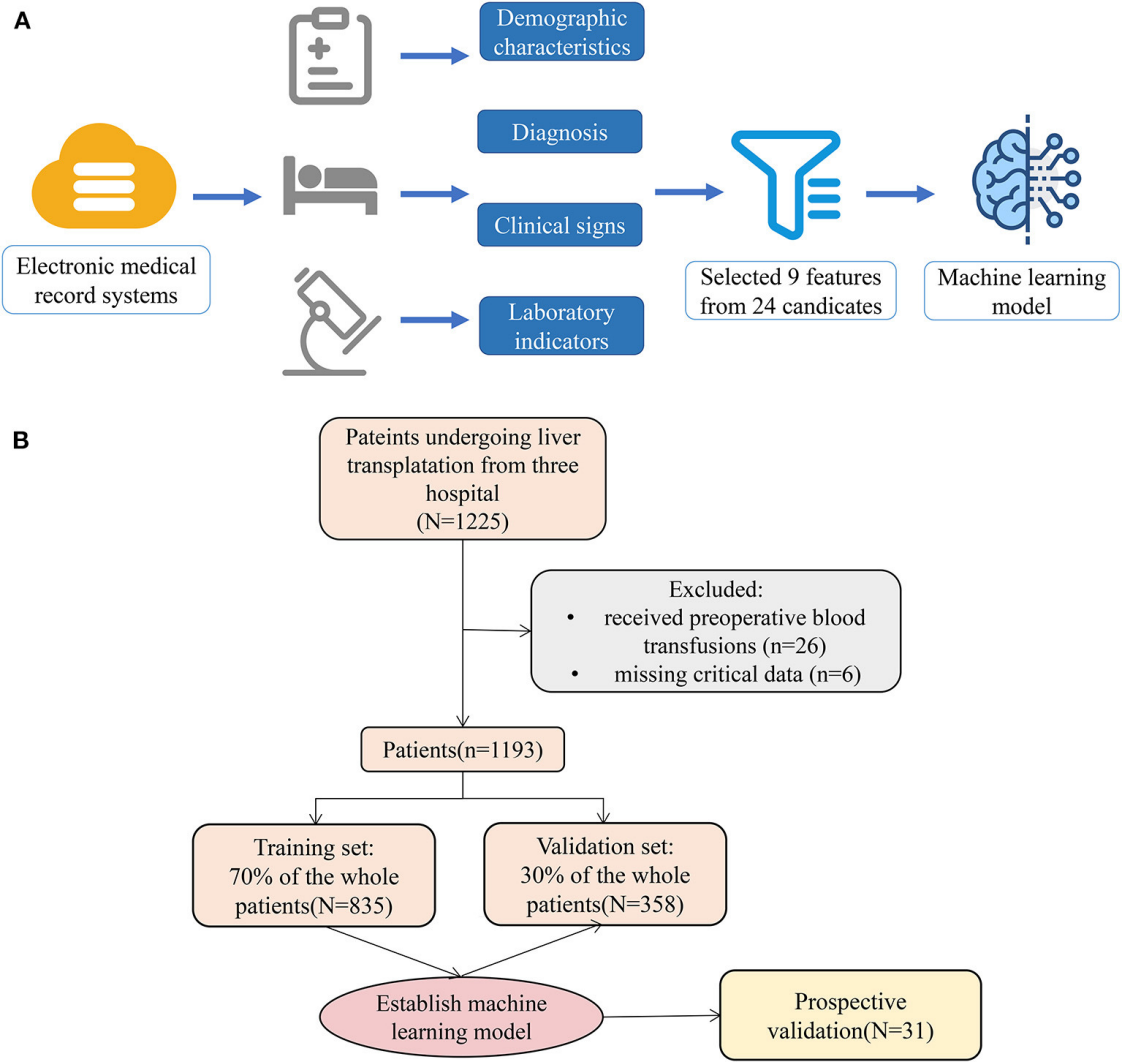
Model-making process and flowchart of the study. **(A)** This figure demonstrated that the data were obtained from the electronic medical record systems of the three hospitals, and all variables included demographic characteristics, diagnosis, clinical signs, and laboratory indicators. A total of 24 preoperative variables were collected, and 9 variables were screened. Moreover, the study used the 9 variables to establish a machine learning model. **(B)** The flowchart of our study.

As shown in Figure 1, 1,193 patients were finally included in this study; the preoperative information of the cohort is shown in Table 1. The average age of patients was 46.17 years, men accounted for 82.73%, and the average weight was 64.15 kg.

**Table 1:** Preoperative information.

**Variable**		**All** **(*n* = 1,193)**	**Non-transfusion group** **(*n* = 329)**	**Transfusion group** **(*n =* 684)**	***p-*value**
Age, mean (SD)		46.17 (11.76)	44.38 (13.95)	46.86 (10.73)	0.004
Sex, *n* (%)	Male	206 (17.27)	60 (18.24)	146 (16.90)	0.645
	Female	987 (82.73)	269 (81.76)	718 (83.10)	0.646
Diagnosis, *n* (%)	Cirrhosis	150 (17.34)	43 (24.02)	107 (15.60)	<0.001
	Liver malignant tumor	154 (17.80)	37 (20.67)	117 (17.06)	<0.002
	Liver failure	83 (9.60)	28 (15.64)	55 (8.02)	<0.003
	Alcoholic hepatitis	42 (4.86)	2 (1.12)	40 (5.83)	<0.004
	Viral hepatitis	257 (29.71)	19 (10.61)	238 (34.69)	<0.005
	Cholestatic liver disease	24 (2.77)	5 (2.79)	19 (2.77)	<0.006
	Others	155 (17.92)	45 (25.14)	110 (16.03)	<0.007
Portal hypertension, *n* (%)		340 (28.50)	43 (13.07)	297 (34.38)	<0.002
Hepatic encephalopathy, *n* (%)		136 (11.40)	201(6.38)	115 (13.31)	0.002
Ascites, *n* (%)		390 (32.69)	64 (19.45)	326 (37.73)	<0.002
Weight, mean (SD)		64.15 (13.22)	62.94 (16.33)	64.43 (12.39)	0.323
ALB, mean (SD)		34.76 (6.16)	35.06 (5.70)	34.68 (6.27)	0.476
ALT, median (Q1, Q3)		53.60 (26.90, 154.90)	51.50 (31.10, 100.90)	54.00 (26.00, 170.00)	0.609
APTT, mean (SD)		51.15 (20.09)	46.06 (13.00)	52.32 (21.22)	<0.001
AST, median (Q1, Q3)		72.00 (38.80, 197.10)	76.60 (40.40, 161.60)	72.00 (38.30, 201.38)	0.527
CR, median (Q1, Q3)		67.00 (55.85, 89.00)	64.00 (56.08, 78.17)	67.80 (55.60, 92.00)	0.035
DBIL, median (Q1, Q3)		67.75 (15.83, 230.18)	29.60 (11.78, 198.00)	84.45 (17.90, 240.70)	0.001
GLO, mean (SD)		26.94 (8.78)	29.39 (7.54)	26.43 (8.94)	<0.001
HB, mean (SD)		102.38 (25.19)	112.30 (29.25)	99.97 (23.51)	<0.001
INR, median (Q1, Q3)		1.63 (1.29, 2.29)	1.46 (1.17, 1.94)	1.67 (1.32, 2.37)	<0.001
PLT, median (Q1, Q3)		69.00 (42.00, 104.50)	87.00 (53.00, 123.00)	66.00 (41.00, 101.00)	<0.001
PT, median (Q1, Q3)		18.90 (15.20, 25.20)	17.20 (14.30, 22.02)	19.25 (15.40, 26.20)	0.002
TBIL, median (Q1, Q3)		105.20 (33.50, 378.27)	51.10 (23.40, 298.70)	135.40 (35.80, 395.80)	0.001
TP, median (Q1, Q3)		61.50 (54.80, 68.25)	65.00 (59.10, 71.20)	60.40 (54.35, 67.30)	<0.001
TT, median (Q1, Q3)		19.50 (17.40, 22.20)	17.80 (16.50, 19.75)	19.80 (17.60, 23.00)	<0.001
UA, median (Q1, Q3)		225.90 (135.05, 332.57)	252.20 (157.12, 339.55)	220.00 (131.25, 332.00)	0.093
Urea, median (Q1, Q3)		5.45 (3.87, 8.10)	5.00 (3.68, 6.71)	5.69 (3.91, 8.50)	0.009
WBC, median (Q1, Q3)		5.21 (3.42, 8.08)	5.58 (3.51, 7.32)	5.21 (3.42, 8.23)	0.972

*SD, standard deviation; ALT, alanine aminotransferase; APTT, activated partial thromboplastin time; AST, aspartate aminotransferase; DBil, Direct bilirubin; PLT, platelet; INR, International standard ratio; PT, prothrombin time; TT, thrombin time; TBil, total bilirubin; WBC, white blood cell*.

Data of 835 patients were used as the training set for model building, and data of 358 patients were used as the validation set. In the training set, 602 (72.1%) patients received RBC transfusion during or after the surgery, and 233 patients did not receive RBC transfusion. In the validation set, 262 (73.2%) patients received RBC transfusion during or after the surgery, and 96 patients did not receive RBC transfusion.

### Key Variables

The nine preoperative variables, including portal hypertension, age, hemoglobin, diagnosis, direct bilirubin, activated partial thromboplastin time (APTT), globulin, aspartate aminotransferase (AST), and alanine aminotransferase (ALT), were selected as crucial variables using the RFE algorithm. As expected, patients with portal hypertension, older age, lower preoperative hemoglobin and globulin levels, approximately longer preoperative APTT, and higher preoperative direct bilirubin, AST, and ALT were more likely to receive RBC transfusion.

**Figure 2:**
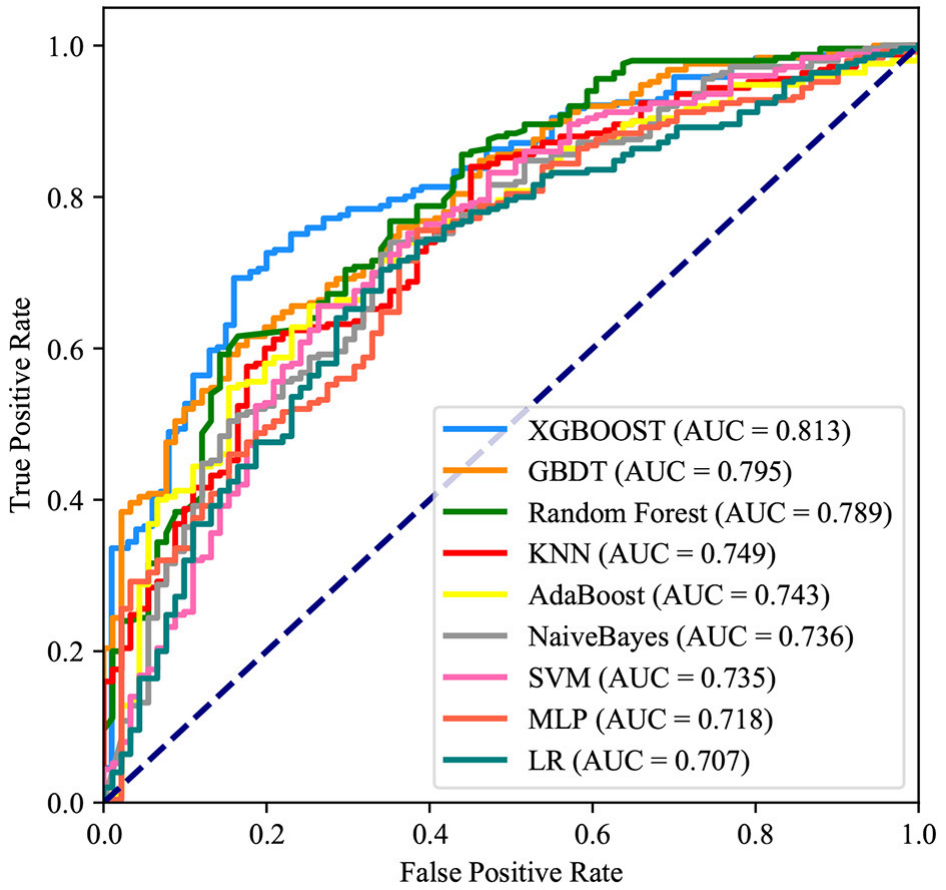
Receiver operating characteristic curves for the machine learning model and logistic regression. XGBOOST, eXtremely Gradient Boosting; GBDT, Gradient Boosting Decision Tree; KNN, K-Nearest Neighbor; SVM, Support Vector Machine; MLP, Multi-Layer Perceptron; LR, Logistic Regression.

After identifying these nine variables, machine learning was used to predict RBC transfusion during or after liver transplantation. As shown in Figure 2, the AUROC of the proposed model was 0.813. The proposed model significantly outperformed the conventional LR (AUROC: 0.707) and seven other machine learning models. According to the Youden Index, defined as sensitivity + specificity – 1, the best cutoff of prediction probabilities of the proposed model was 0.737 (shown in Table 2), with a sensitivity and specificity of 66.4 and 85.0%, respectively. The best cutoff of prediction probabilities of LR was 0.626, with a sensitivity and specificity of 70.4 and 65.9%, respectively.

**Table 2:** Analysis of sensitivity and specificity.

**Model**	**Best cutoff**	**Accuracy**	**Youden Index**	**Sensitivity**	**Specificity**	**PPV**	**NPV**
XGBOOST	0.737	0.718	0.514	0.664	0.850	0.914	0.512
GBDT	0.803	0.672	0.440	0.616	0.824	0.906	0.439
Random Forest	0.790	0.674	0.451	0.616	0.835	0.911	0.442
KNN	0.763	0.660	0.403	0.612	0.791	0.890	0.426
AdaBoost	0.507	0.680	0.403	0.656	0.747	0.877	0.442
NaiveBayes	0.124	0.716	0.388	0.740	0.648	0.853	0.476
SVM	0.743	0.677	0.392	0.656	0.736	0.872	0.438
MLP	0.631	0.718	0.371	0.756	0.615	0.844	0.479
LR	0.626	0.692	0.363	0.704	0.659	0.850	0.448

*The best cutoff was determined by Youden index, defined as sensitivity + specificity – 1. XGBOOST, eXtremely Gradient Boosting; GBDT, Gradient Boosting Decision Tree; KNN, K-Nearest Neighbor; SVM, Support Vector Machine; MLP, Multi-Layer Perceptron; LR, Logistic Regression; PPV, Positive Predictive Value; NPV, Negative Predictive Value*.

### Application of the Model

**Figure 3:**
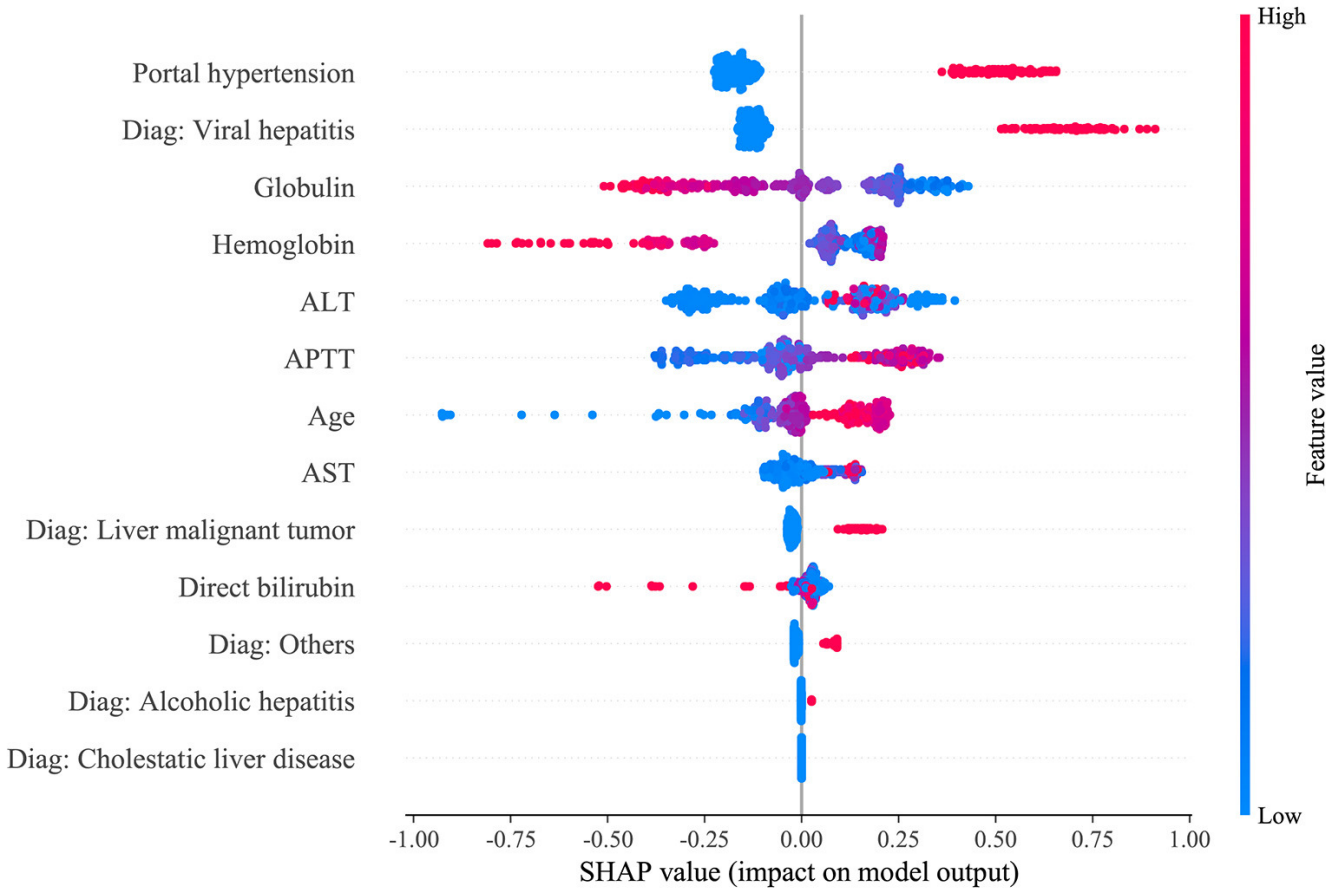
SHAP analysis of the proposed model on the validation set. This figure described data from the validation set, with each point representing one patient. The color represents the value of the variable; red represents the larger value; blue represents the smaller value. The horizontal coordinates represent a positive or negative correlation with transfusion risk, with a positive value indicating a risk of transfusion and a negative value indicating no need for transfusion. The absolute value of the horizontal coordinate indicates the degree of influence; the greater the absolute value of the horizontal coordinate, the greater the degree of influence.

**Figure 4:**
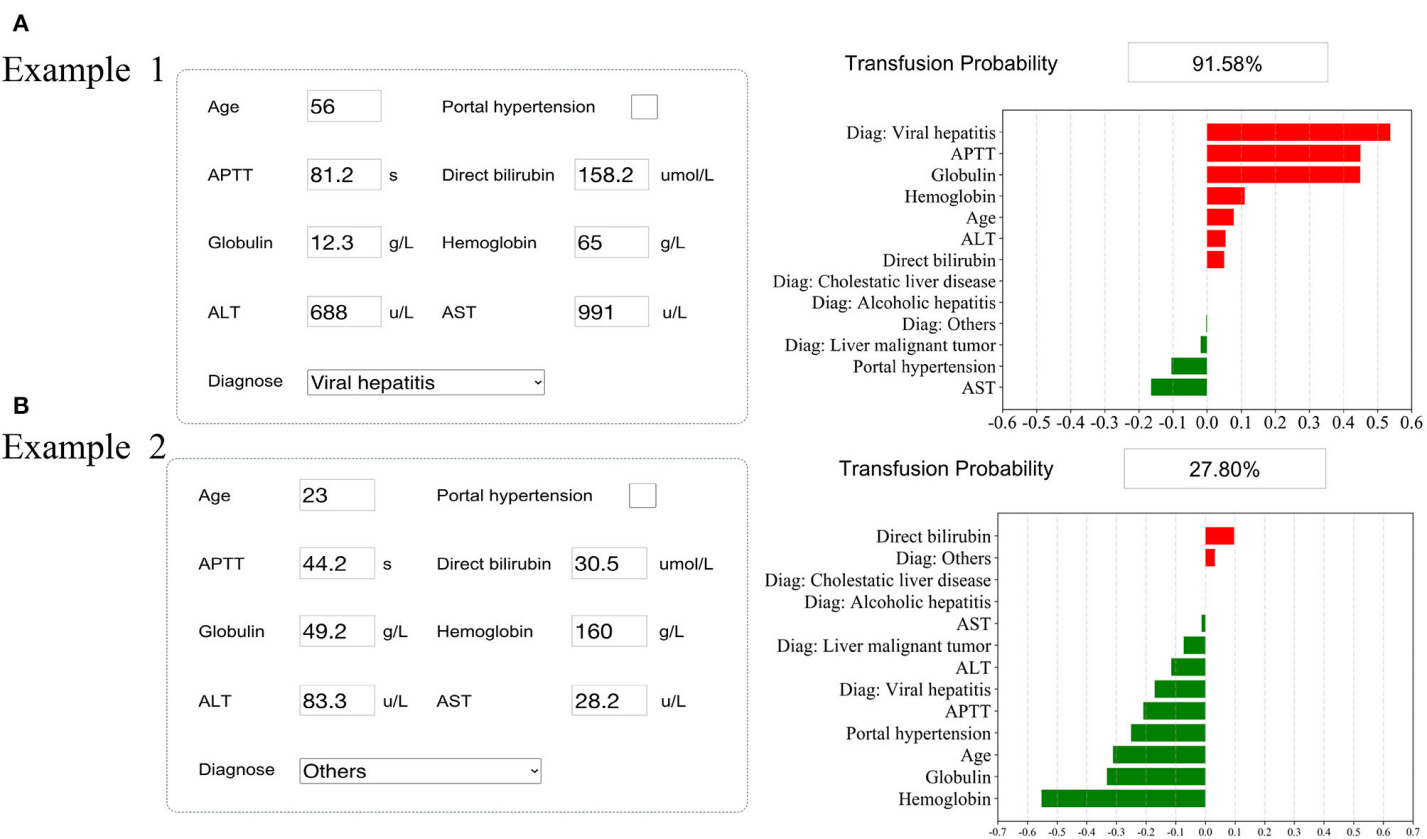
Examples of website usage. Entering the input value determined the transfusion requirements and displayed how each value contributed to the prediction. **(A)** Example 1 needs RBC transfusion, and **(B)** Example 2 does not need RBC transfusion.

The SHAP package analyzed the entire training set, showing the impact of each variable on predicting transfusion (Figure 3). The preoperative information of a patient was input into the model: age 56 years, no portal hypertension, diagnosed with viral hepatitis, hemoglobin 65 g/L, direct bilirubin level 158.2 μmol/L, APTT 81.2 s, globulin level 12.3 g/L, ALT 688 U/L, and AST 991 U/L. The model analyzed that the risk of RBC transfusion in this patient was 91.58%, indicating that the probability of RBC transfusion for the patients was high, and RBC transfusion was recommended (Figure 4A). The preoperative information of another patient was input into the model: age 23 years, no portal hypertension, diagnosed with other disease, hemoglobin 160 g/L, direct bilirubin 30.5 μmol/L, APTT 44.2 s, globulin 49.2 g/L, ALT 83.3 U/L, and AST 28.2 U/L. The predicted probability of transfusion in this patient was 27.80%, indicating that the patient was at low risk of needing an RBC transfusion (Figure 4B). Furthermore, a website was established for clinicians to use the proposed model, http://www.aimedicallab.com/tool/aiml-livertrans.html.

### Prospective Validation

Data of 31 patients were prospectively collected for validation, of which 87% (25) were transfused during or after liver transplantation surgery. The accuracy of the proposed model on the prospective dataset was 76.9%. There was one patient who was transfused but whom the model predicted as negative. He had an accidental intraoperative hemorrhage (about 2,000 ml of blood loss). In the eight patients who were nontransfused but whom the model predicted as positive, two was transfused with a large number of platelets and the others had probabilities (75.41–83.49%) close to the cutoff.

## Discussion

This study was novel in using machine learning algorithms to predict RBC transfusion during or after liver transplantation. A machine learning model was built that could accurately predict RBC transfusion during or after liver transplantation before the surgery, better than other models developed in this study. The model established in this study had great discrimination and showed satisfactory specificity and sensitivity. Therefore, the hypothesis proposed in this study was supported by the results.

Several studies showed that RBC transfusion increased complications and was related to a lower 5-year survival rate (3, 26). In addition, costs associated with transfusing a single unit of blood were significantly high, including the cost of treating any adverse effect of transfusion or the associated increased length of hospital stay. These costs far outweighed the lower cost of the use of tranexamic acid, erythropoietin (EPO), oral treatments of anemia, intravenous iron therapy, and cell salvage utilization. As a result, clinicians have taken many measures to reduce RBC transfusions (25, 27). By predicting RBC transfusion before surgeries, high-risk patients could be identified. The management of patients could be improved, thus improving outcomes and reducing morbidity and cost (4, 28, 29). Therefore, it was of great importance to predict RBC transfusion before surgeries and take corresponding preoperative measures.

In this study, a machine learning model was developed to predict RBC transfusion, which could help clinicians identify high-risk patients. If the model identified patients at low probability of transfusion, potentially unnecessary repeat testing was exempt, such as a complete blood count or further preoperative laboratory testing. Therefore, this model might be a valuable tool to avoid wasteful and unnecessary medical tests. Alternatively, identifying patients at high risk for transfusion might improve the efficiency of perioperative blood management and reduce transfusions. It was suspected that for each transfusion avoided, the patient and financial benefit might be significant due to the large number of patients undergoing gynecologic surgery. Future investigations should include measuring the model's impact on patient and cost outcomes.

In addition, two examples were used to visualize how the model could predict RBC transfusion and determine the relative importance of each variable for the clinician. With millions of liver transplants taking place each year, the findings could help surgeons perform liver transplants, while also giving patients information about their probabilities of receiving RBC transfusion before surgery.

Previous studies reported that intraoperative blood loss and postoperative decreased hemoglobin levels were associated with the risk of receiving an RBC transfusion (30–32). However, preoperative information should be used to predict the need for RBC transfusion so as to find other risk features; otherwise, it is too late to take action to determine transfusion risk through intraoperative or postoperative information.

The significance of this study was that it combined preoperative characteristic variables other than hemoglobin to establish a clinical prediction model. Portal hypertension, age, hemoglobin, diagnosis, direct bilirubin, APTT, ALT, AST, and globulin were selected as important variables. Arshad found that portal hypertension was associated with increased blood loss and RBC transfusion in orthotopic liver transplantation (33), which was similar to the result of the present analysis. Fabio Bagante established a nomogram of hepatectomy to predict the risk of transfusion and included total bilirubin among the risk factors for transfusion. However, the present study found that the level of direct bilirubin correlated with the risk of transfusion in patients undergoing liver transplantation (34). Most studies assessing the risk of transfusion also demonstrated a vital role for age and preoperative hemoglobin in predicting transfusion (3, 35, 36). All of the aforementioned studies supported the results of the present study very well. Besides, this study also found other variables that increased the risk of RBC transfusion, including preoperative APTT, AST, ALT, and globulin. APTT reflects the patient's coagulation function; the lower the coagulation function, the greater the likelihood of intraoperative blood loss, thus increasing the risk of RBC transfusion. Therefore, clinical decision-makers should consider using the pro-coagulation treatment and administering drugs that could alter the coagulation state with careful thinking for patients predicted as high-risk groups. An abnormal level of AST, ALT, or globulin reflected the poor state of a patient's liver function, which might indirectly represent a decreased coagulation state and increased risk of transfusion. Focusing solely on hemoglobin to determine whether to transfuse might be of limited utility, and comprehensive inclusion of preoperative patient information could help guide clinical transfusion decisions and more effective blood management. For high-risk patients, clinicians should consider correcting hemoglobin before surgery and provide liver protection treatment to improve liver function, coagulation function, and portal hypertension.

In this study, an RBC transfusion prediction model was developed with great discrimination. This study included multi-center datasets and prospective validation, which was also an advantage compared with other studies; the abundant data allowed rigorous evaluation of the performance of machine learning models. Ultimately, the approach used in the present study can be applied to a variety of problems that arise before and after surgery to make the surgery safe. Furthermore, it can also be applied to other complications and operations, such as sepsis and acute kidney injury (37–41).

This study had several limitations. First, the transfusion criteria were not the same in each institution; therefore, the definition of the transfusion group was different. A vast majority of institutions were based on a restrictive transfusion strategy, where patients were transfused when their hemoglobin was <70 g/L (42, 43). Second, the surgeons at each institution had different surgical plans; other factors might also lead to blood transfusions, thus affecting the results. Third, the training and the validation sets were divided as a 7:3 ratio, and using other external validation sets might yield different results. Therefore, more datasets from other centers were needed for validation. Fourth, patients with missing critical data were excluded, causing selection bias. Fifth, like other retrospective studies, a selection bias might exist without considering unknown confounding factors. Lastly, although SHAP values were used to help interpret our machine learning model, a more interpretable model is still needed in clinical practice (44). As a future work, we planned to develop a Nomogram or machine learning-based automatic clinical scoring system based on our data, in order to provide clinicians a more usable and easy-to-understand tool (45).

## Conclusions

In this study, a machine learning algorithm was used to develop an RBC transfusion prediction model during and after liver transplantation, which was expedient and had good performance. This model could realize the individualized prediction of RBC transfusion and minimize the cost and risk of various blood transfusion preventive measures. The study recommended using this model to predict RBC transfusion before liver transplantation and instruct high-risk patients to take appropriate preventive measures. A prospective blood management database should be built to minimize selection bias, machine learning models should be developed based on the preoperative characteristics of patients undergoing liver transplantation, and the models should be validated with data from such patients in the future. Finally, a randomized controlled trial should be conducted to evaluate the impact of machine learning models, as decision supporters for clinicians, on clinician behavior, healthcare utilization, and patient outcomes.

## Data Availability Statement

The original contributions presented in the study are included in the article/supplementary material, further inquiries can be directed to the corresponding author/s.

## Ethics Statement

The present study did not intervene in liver transplantation surgeries. That means the study did not decide who and when to undergo surgery, who to be the donors, and when to transfuse RBCs. They were all carried out according to the standard procedure at the three hospitals. Patients' clinical data were collected, and the model was developed. None of their identifiable data were collected. The study was approved by local ethics committees.

## Author Contributions

L-PL, Q-YZ, and RG designed and performed the study. L-PL, HD, Z-WC, Y-JW, and JW collected the data. Q-YZ performed the analytic calculations and statistical analysis. L-PL and Q-YZ wrote the manuscript, which was improved under the guidance of Y-WL. All authors provided critical feedback and helped to shape the research, analysis, and manuscript.

## Conflict of Interest

The authors declare that the research was conducted in the absence of any commercial or financial relationships that could be construed as a potential conflict of interest.
